# Effect of High Myopia on Cataract Surgery: An Age-Matched Case-Control Study

**DOI:** 10.7759/cureus.101596

**Published:** 2026-01-15

**Authors:** Xin Gen Ng, Jessica Mani Penny Tevaraj, Shahrul Aiman Soelar, Ang Ee Ling

**Affiliations:** 1 Ophthalmology, Hospital Pulau Pinang, George Town, MYS; 2 Clinical Research Centre, Hospital Sultanah Bahiyah, Alor Setar, MYS

**Keywords:** cataract surgery, high myopia, ocular axial length, ocular biometry, spherical equivalent

## Abstract

Introduction

This study aimed to investigate the demographic factors and outcomes of cataract surgery in patients with high myopia.

Methods

This was an age-matched case-control study conducted at Hospital Pulau Pinang, Malaysia, involving patients who underwent phacoemulsification with intraocular lens implantation between September 2022 and August 2023. Seventy patients with axial length (AXL) ≥ 26.00 mm were included in the high myopia group, while 70 age-matched patients with AXL 22.00-25.99 mm served as the control group.

Results

The mean age of both groups was 67.3 years (SD = 7.86). Significant differences were observed between the control and high myopia groups in sex (p = 0.009), ethnicity (p < 0.001), AXL (p < 0.001), keratometric reading (K1) (p = 0.033), biometry (p = 0.049), and preoperative targeted spherical equivalent (SE) (p < 0.001). In the high myopia group, laser interferometry achieved a significantly higher proportion of postoperative SE within ±1.00 D compared with immersion ultrasound (p = 0.043), while no significant difference was found between biometry methods in the control group (p = 1.000). No significant differences were observed between the groups in preoperative or postoperative best corrected visual acuity (BCVA), BCVA improvement thresholds (≥ 0.2, ≥ 0.4, and ≥ 0.6 logMAR), K2, achieved SE, or SE differences. Simple and multiple logistic regression performed as a secondary analysis did not identify any significant predictors of postoperative SE > ±1.0 D.

Conclusion

Male sex and Chinese ethnicity were associated with high myopia in this cohort. In highly myopic eyes, the use of laser interferometry was associated with better refractive outcomes compared to immersion biometry. Surgeons intentionally targeted a more myopic preoperative SE in highly myopic eyes as a deliberate refractive strategy to reduce the risk of hyperopic surprise and to achieve postoperative refractive outcomes comparable to the control group.

## Introduction

High myopia is a common refractive error characterized by a high degree of nearsightedness and is defined as an axial length (AXL) ≥ 26 mm or a spherical equivalent (SE) ≤ -6.00 diopters [1]. The incidence of myopia is increasing worldwide, especially in Asian populations [2].

These eyes frequently have degenerative changes involving the sclera, choroid, vitreous, and retina. High myopia has been identified as a risk factor for cataract formation [3]. All of these factors can potentially impact the outcomes of cataract surgery. Cataract surgery in highly myopic eyes is relatively complex and challenging for ophthalmologists because of its association with difficulty in estimating the visual outcome and poor fundus condition [4].

High myopia has been associated with educational level [5], and individuals with high myopia tend to develop cataracts approximately 10 years earlier than eyes with normal AXL [6]. This group often has higher expectations regarding postoperative visual outcome. Therefore, understanding the effects of high myopia on cataract surgery outcomes is important for optimizing patient care and managing the unique challenges presented by this specific patient population.

This study aimed to investigate the demographic factors and outcomes of cataract surgery in highly myopic individuals. High myopia was defined as an AXL ≥ 26 mm, while the control group included eyes with AXL between 22 and 25.99 mm. AXL was chosen as the parameter, as nuclear cataract alone may induce refractive myopia with normal AXL and keratometric value [3].

The results of this study may provide practical implications for surgical planning and patient counselling. Identifying differences in refractive outcomes may help tailor perioperative planning and set appropriate expectations for patients with high myopia. Data from routine public hospital practice describing demographic factors, refractive outcome, biometry use, and intentional refractive targeting in highly myopic patients remain limited. This study, therefore, aims to evaluate these components between highly myopic patients compared with age-matched controls.

## Materials and methods

Study design and participants

This was a retrospective age-matched case-control study conducted at Hospital Pulau Pinang. Cases and controls were matched in a 1:1 ratio based on age, with no additional variables included. The study population included patients who underwent phacoemulsification with posterior chamber intraocular lens (IOL) implantation between 1 September 2022 and 31 August 2023. Inclusion criteria were patients aged 18 years and older with complete preoperative and postoperative refraction and biometry data, with preoperative targeted SE aimed at achieving postoperative emmetropia.

Exclusion criteria included traumatic cataract, previous ocular surgery (including vitreoretinal, glaucoma, or refractive surgery), and ocular conditions that could affect refractive accuracy or visual outcomes, such as severe corneal scarring or opacities, diabetic retinopathy, diabetic macular oedema, age-related macular degeneration, myopic choroidal neovascularization, and advanced glaucoma.

Ophthalmic examinations and definitions

Patients' data were retrieved from the National Eye Database (NED) Malaysia. Ocular biometry was performed using the IOLMaster 500 (Carl Zeiss Meditec AG, Jena, Germany) and the PacScan Plus 300A+ immersion A-scan ultrasonography system (Sonomed Escalon, Lake Success, NY, USA). IOL power calculation was performed using SRK/T formula. Visual acuity was assessed using a logarithm of the minimum angle of resolution (logMAR) chart for all patients.

Postoperative best corrected visual acuity (BCVA) for all the patients was evaluated at six weeks, as this reflects routine clinical practice at our institution, allowing sufficient time for postoperative healing and refractive stabilization. Postoperative refractive outcomes were categorised based on SE, with an SE within ±1.0 dioptre considered an acceptable refractive outcome, and an SE greater than ±1.0 dioptre considered a refractive error.

Statistical analysis

The findings were analyzed using IBM SPSS Statistics for Windows, Version 24 (Released 2016; IBM Corp., Armonk, New York, United States). Descriptive statistics were presented as frequencies (percentages) for categorical data, as means (standard deviations) for normally distributed numerical data, and as medians (interquartile ranges) for non-normally distributed numerical data. Cases and controls were age-matched in a 1:1 ratio based on patient’s age. After matching, the independent t-test was employed to analyze the differences in normally distributed numerical data between two independent groups, whereas the Mann-Whitney test was utilized for non-normally distributed data. Pearson's chi-square test for independence was employed to examine the relationship between two sets of categorical data, while Fisher’s exact test was used when expected cell counts were less than 5. Normality was assessed at the variable level within each group using skewness and kurtosis (acceptable ranges: -1 to 1 and -3 to 3, respectively), with visual inspection of distributions performed to confirm the appropriateness of parametric testing.

Subsequently, simple and multiple logistic regression analyses were performed to evaluate the secondary outcome, which was the association between potential risk factors and postoperative refractive outcome. The independent variables were entered using the enter method, as all variables were included based on clinical relevance rather than automated selection criteria. Model fit was assessed using the Hosmer-Lemeshow goodness-of-fit test. Multicollinearity was assessed using the variance inflation factor (VIF), and a VIF less than 10 was considered no significant multicollinearity [7]. Results were reported as odds ratios (ORs) with corresponding 95% confidence intervals (CIs). Given the relatively small number of refractive outliers, the logistic regression analysis may have limited statistical power, and results should be interpreted with caution. All probability values were two-sided, and a p-value < 0.05 was considered statistically significant.

## Results

Matching variable

A total of 70 patients with high myopia (mean (SD) age = 67.3 (7.86) years) were included in the high myopia group. Seventy age-matched controls with normal AXL were included in the control group. Since age was used as a matching variable, there was no observed difference in age distribution between the groups, ensuring that the analysis of other variables (e.g., sex, ethnicity, AXL, BCVA) is not confounded by age. Table 1 shows the association between variables and high myopia.

**Table 1:** Demographic Factors and Clinical Characteristics of High Myopia and Control Groups ^a ^Pearson’s chi-square test;^ b ^Mann-Whitney test, presented as median (interquartile range); ^c ^Independent t-test, presented as mean (standard deviation) AXL: axial length; K: keratometric reading

Variables	High myopia group		Control group		P-value
Matching variable					
Age (years), mean (SD)	67.3	(7.9)	67.3	(7.9)	-
Sex, n (%)					
Male	34	(48.6)	19	(27.1)	0.009^a^
Female	36	(51.4)	51	(72.9)	
Ethnicity, n (%)					
Chinese	66	(94.3)	41	(58.6)	<0.001^a^
Malay	3	(4.3)	16	(22.9)	
Indian	1	(1.4)	12	(17.1)	
Others	0	0.0	1	(1.4)	
Eye, n (%)					
Left eye	26	(37.1)	34	(48.6)	0.172^a^
Right eye	44	(62.9)	36	(51.4)	
AXL, median (IQR)	26.9	(1.6)	23.5	(0.8)	<0.001^b^
K1, mean (SD)	43.4	(1.7)	44.0	(1.2)	0.033^c^
K2, mean (SD)	44.4	(1.7)	44.9	(1.2)	0.069^c^

Demographic interpretation

Male participants were more frequently represented in the high myopia group compared with the control group (48.6% vs. 27.1%), whereas female participants predominated in the control group (72.9%), indicating a significant association between sex and high myopia (p-value = 0.009). Chinese participants were more frequently represented in the high myopia group compared with the control group (94.3% vs. 58.6%), demonstrating a significant association between ethnicity and high myopia (p < 0.001).

Clinical characteristics

Laterality (p-value = 0.172) and K2 (p-value = 0.069) variables did not differ significantly between the control and high myopia groups. In contrast, AXLs (p-value < 0.001) and K1 (p-value = 0.033) showed statistically significant differences between groups. Specifically, mean AXLs were higher in the high myopia group (mean (SD) = 26.9 (1.6)) compared to the control group (mean (SD) = 23.5 (0.8)). K1 was higher in the control group (mean (SD) = 44.0 (1.2)) compared to the high myopia group (mean (SD) = 43.4 (1.7)), as shown in Table 1.

BCVA

**Figure 1:**
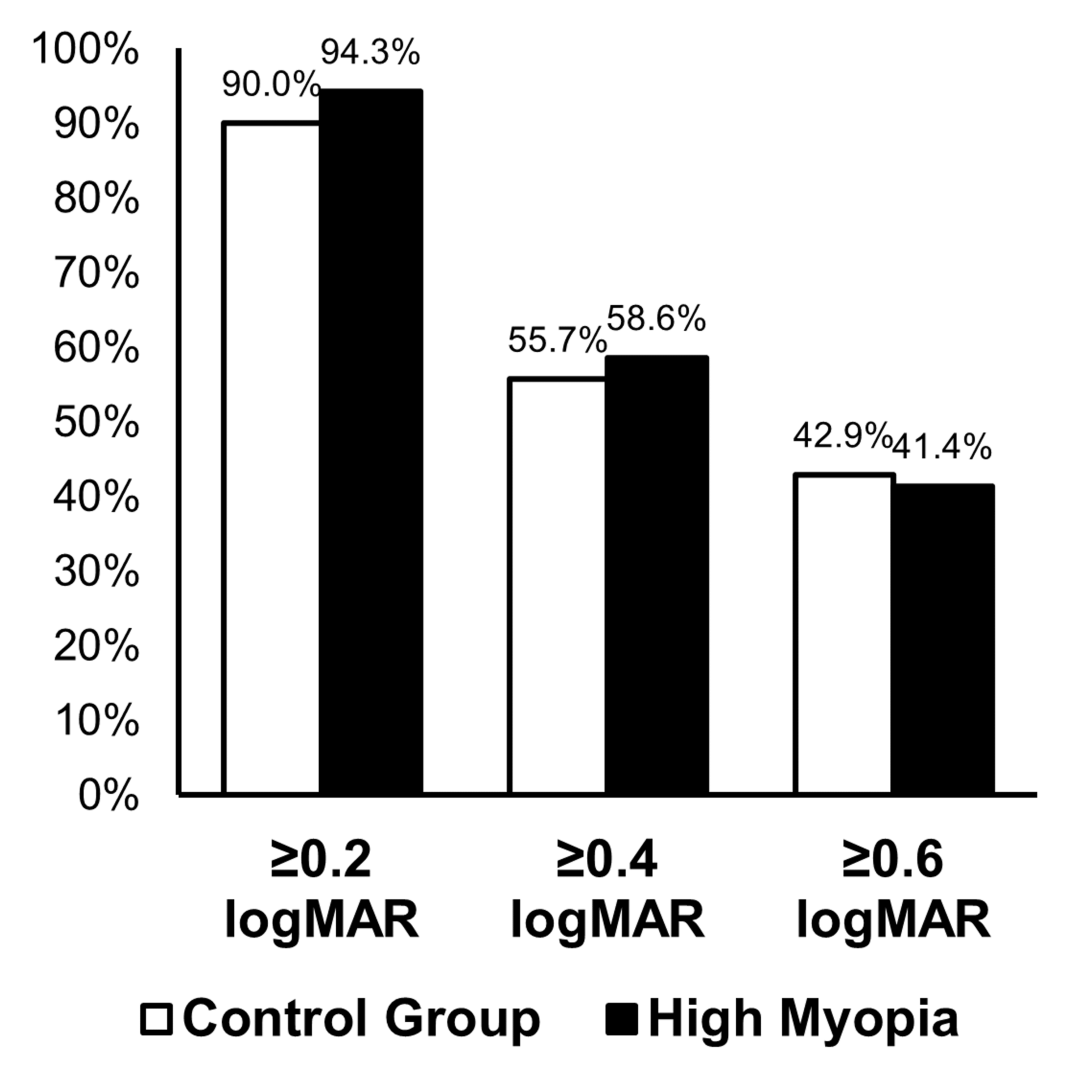
Comparison of BCVA Improvement Between High Myopia and Control Groups BCVA: best corrected visual acuity

There was no significant difference in preoperative BCVA (p-value = 0.743) and postoperative BCVA (p-value = 0.761). BCVA improvements between the high myopia and control group did not differ significantly in achieving ≥0.2 (p-value = 0.346), ≥0.4 (p-value = 0.733), and ≥ 0.6 logMAR (p-value = 0.865), respectively, as shown in Table 2 and better illustrated in Figure 1.

**Table 2:** Comparison of Preoperative and Postoperative BCVA Improvement Between High Myopia and Control Groups ^a ^Mann-Whitney test, presented as median (interquartile range); ^b ^Pearson’s chi-square test BCVA: best corrected visual acuity

BCVA improvement	High myopia group		Control group		P-value
Preoperative BCVA, median (IQR)	0.6	(0.7)	0.6	(0.5)	0.743^a^
Postoperative BCVA, median (IQR)	0.2	(0.2)	0.2	(0.2)	0.761^a^
BCVA improvement, n (%)					
≥0.2 logMAR	66	(94.3)	63	(90.0)	0.346^b^
≥0.4 logMAR	41	(58.6)	39	(55.7)	0.733^b^
≥0.6 logMAR	29	(41.4)	30	(42.9)	0.865^b^

Biometry

**Figure 2:**
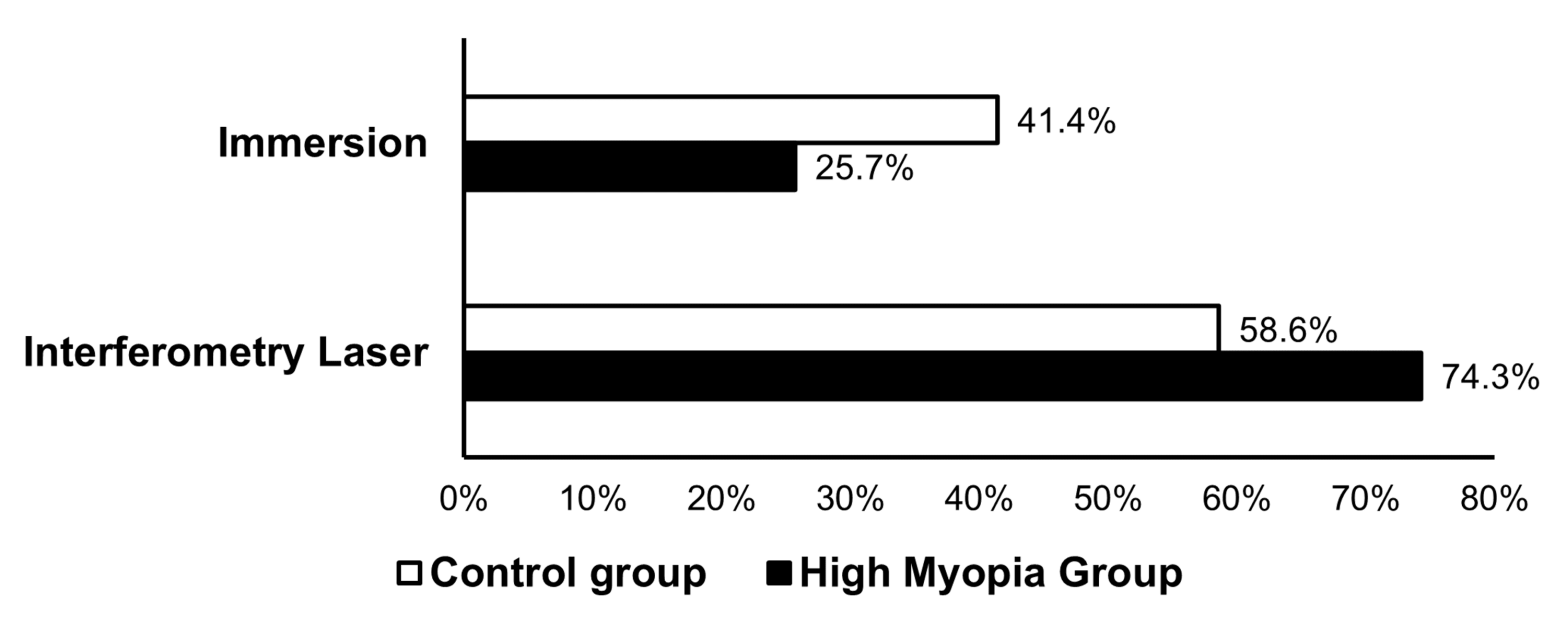
Method of Biometry Used (Laser Interferometry Versus Immersion) in the Control and High Myopia Groups

Laser interferometry was the preferred method of biometry compared with immersion, with a statistically significant difference observed (p-value = 0.049) based on Pearson’s chi-square test (Figure 2). A total of 52 patients (74.3%) in the high myopia group underwent laser interferometry, compared with 41 patients (58.6%) in the control group. In the high myopia group, laser interferometry achieved a refractive outcome of SE ≤ 1.00 D in 92.3% of cases, significantly outperforming immersion ultrasound, which achieved the same target in only 72.2% (p-value = 0.043). Whereas in the control group, there was no significant difference between laser interferometry and immersion in achieving a refractive outcome of ≤ 1.00 D (p-value = 1.000), as shown in Table 3.

**Table 3:** Association Between Biometry Method and Postoperative Refractive Outcome in High Myopia and Control Groups ^a ^Fisher’s exact test SE: spherical equivalent

Group	Biometry Method	Refractive outcome SE ≤ ±1D		Refractive outcome SE > ±1D		P-value
		n	(%)	n	(%)	
High myopia	Laser interferometry	48	(92.3)	4	(7.7)	0.043^a^
	Immersion	13	(72.2)	5	(27.8)	
Control group	Laser interferometry	38	(92.7)	3	(7.3)	1.000^a^
	Immersion	27	(93.1)	2	(6.9)	

Preoperative and postoperative targeted SE

Table 4 illustrates a comparison between the preoperative targeted SE and the postoperative achieved SE for both the control and high myopia groups. The mean preoperative targeted SE was less negative in the control group (mean (SD) = -0.4 (0.11)) compared to the high myopia group (mean (SD) = -0.5 (0.17)) (p-value < 0.001). Postoperative achieved SE (p-value = 0.702), and SE difference (p-value = 0.394) did not show significant differences between groups. Refractive outcome of SE ≤ ±1D between the high myopia and control group does not show significant differences as well (p-value = 0.260).

**Table 4:** Comparison of Preoperative and Postoperative SE and Refractive Outcomes Between High Myopia and Control Groups ^a^ Independent t-test, presented as mean (standard deviation);^ b ^Mann-Whitney test, presented as median (interquartile range);^ c ^Pearson’s chi-square test SE: spherical equivalent

Variables	High myopia group		Control group		P-value
Preoperative targeted SE, mean (SD)	-0.5	(0.2)	-0.4	(0.1)	<0.001^a^
Postoperative achieved SE, mean (SD)	-0.5	(0.6)	-0.5	(0.5)	0.702^a^
SE difference, median (IQR)	0.3	(0.4)	0.3	(0.4)	0.394^b^
Refractive outcome, n (%)					
SE ≤ ±1D	61	(87.1)	65	(92.9)	0.260^c^
SE > ±1D	9	(12.9)	5	(7.14)	

Logistic regression analyses of factors affecting postoperative refractive outcome

A total of 140 eyes were included, comprising 70 high myopia eyes and 70 age-matched controls. Overall, 126 eyes (90.0%) achieved a postoperative SE within ±1.0 D, while 14 eyes (10.0%) were outside this range.

In simple logistic regression, age was not significantly associated with postoperative refractive error (SE > ±1.0D) (crude odds ratio (COR) 0.98; 95% CI 0.92, 1.05; p = 0.550). Female sex showed higher odds of postoperative SE greater than ±1.0 D compared to males, although this difference was not statistically significant (COR 1.59; 95% CI 0.47, 5.35; p = 0.453). Similarly, ethnicity did not demonstrate a significant association, with other ethnic groups showing refractive outcomes comparable to Chinese patients (COR 0.87; 95% CI 0.23-3.34; p = 0.842). Eyes with high AXL (≥26 mm) had higher odds of achieving SE > ±1.0 D compared with control eyes, but this association did not reach statistical significance (COR 1.92; 95% CI 0.61, 6.04; p = 0.266). Biometry method showed no significant association with refractive outcome, although immersion ultrasound demonstrated a non-significant trend toward increased odds of postoperative refractive error (SE > ±1.0 D) (COR 2.15; 95% CI 0.71, 6.54; p = 0.178).

In the multiple logistic regression model adjusting for age, sex, ethnicity, AXL group, and biometry method, none of the variables demonstrated statistically significant associations with refractive error. Age remained non-significant (adjusted odds ratio (AOR) 0.98; 95% CI 0.92, 1.05; p = 0.604), and the association for female sex continued to be non-significant (AOR 1.96; 95% CI 0.56-6.81; p = 0.291). Ethnicity showed no independent effect on refractive error (AOR 1.35; 95% CI 0.28-6.55; p = 0.707). High AXL remained a clinically relevant factor, showing a trend toward increased odds of postoperative refractive error (SE > ±1.0 D); however, this association did not reach statistical significance (AOR 2.82; 95% CI 0.71,11.16; p = 0.141). Biometry modality also did not significantly influence refractive outcomes, with immersion ultrasound demonstrating a non-significant trend toward higher odds of refractive error compared with laser interferometry (AOR 2.43; 95% CI 0.77, 7.72; p = 0.131) (Table 5).

**Table 5:** Simple and Multiple Logistic Regression Analyses of Factors Associated With Postoperative Refractive Outcomes VIF: variance inflation factor; SE: spherical equivalent; COR (95% CI): Crude odds ratio (95% confidence interval); AOR (95% CI): adjusted odds ratio (95% confidence interval) Summary statistics Model test: Hosmer-Lemeshow goodness-of-fit test: χ^2^(df) = 5.22 (5) = 0.390 Nagelkerke’s R^2^ = 0.077

Variables	Refractive outcome				Simple logistic regression			Multiple logistic regression			VIF
	SE ≤ ±1D		SE > ±1D		COR	(95% CI)	P-value	AOR	(95% CI)	P-value	
Age (years), median (IQR)	68.0	(9.00)	68.5	(7.00)	0.98	(0.92,1.05)	0.550	0.98	(0.92,1.05)	0.604	1.01
Sex, n (%)											
Male	49	(92.5)	4	(7.5)	1.00	(ref.)	0.453	1.00	(ref.)	0.291	1.03
Female	77	(88.5)	10	(11.5)	1.59	(0.47,5.35)		1.96	(0.56,6.81)		
Ethnicity, n (%)											
Chinese	96	(89.7)	11	(10.3)	1.00	(ref.)	0.842	1.00	(ref.)	0.707	1.34
Other ethnicity	30	(90.9)	3	(9.1)	0.87	(0.23,3.34)		1.35	(0.28,6.55)		
Axial length group, n (%)											
High myopia	61	(87.1)	9	(12.9)	1.92	(0.61,6.04)	0.266	2.82	(0.71,11.16)	0.141	1.40
Control	65	(92.9)	5	(7.1)	1.00	(ref.)		1.00	(ref.)		
Biometry, n (%)											
Laser interferometry	86	(92.5)	7	(7.5)	1.00	(ref.)	0.178	1.00	(ref.)	0.131	1.04
Immersion	40	(85.1)	7	(14.9)	2.15	(0.71,6.54)		2.43	(0.77,7.72)		

Overall, while high AXL and immersion ultrasound biometry demonstrated a consistent non-significant trend toward postoperative refractive error, none of the evaluated variables showed statistically significant associations with postoperative SE within ±1.0 D. The limited number of refractive outliers may have reduced the statistical power to detect significant associations, and these findings should therefore be interpreted with caution.

## Discussion

Our study found no difference in age distribution between the high myopia and control groups, as age was used as a matching variable to ensure that the analysis of other variables (e.g., sex, ethnicity, BCVA, SE, and refractive outcome) was not confounded by age. The high myopia group had a higher number of male patients compared to the control group, consistent with studies highlighting a gender disparity in myopia prevalence in older age groups [8]. However, the pattern was reversed in younger generations, where females have a higher risk of developing high myopia [8]. A substantially larger proportion of high myopia patients were of Chinese populations, which aligns with previous studies that report a higher prevalence of myopia and high myopia among East Asian populations. This is potentially due to a combination of genetic predisposition and environmental factors such as reduced outdoor time and increased engagement in near-work activities [9,10].

Preoperative BCVA did not differ significantly between the high myopia and control groups (p = 0.743), indicating that both groups had comparable baseline vision before surgery. Postoperative BCVA also showed no significant difference between the two groups (p = 0.761) as well. This suggests that, despite the anatomical challenges associated with longer AXL, cataract surgery can provide similarly favorable visual outcomes in highly myopic patients when retinal pathology is excluded [3,4]. High myopia patients experience comparable visual improvements to the control group after cataract surgery. More than 90% gained at least 0.2 logMAR, and a substantial proportion achieved ≥0.4 and ≥0.6 logMAR improvement. There was no significant difference between groups, meaning high myopes can expect good vision as long as the retina is healthy and biometry is accurate. This finding is consistent with Tan et al. [11], who reported that over 85% of high-myopia patients achieved meaningful visual improvement after cataract surgery, despite the challenges associated with elongated AXL and increased optical aberrations.

Laser interferometry was more frequently used in the high myopia group (74.3%) compared to the control group (58.6%). This preference reflects the well-documented advantages of laser interferometry, which has been reported to provide more accurate AXL measurements in high myopic patients [12]. Among high-myopia patients, laser interferometry achieved postoperative SE ≤1.00 D in 92.3% of cases (48/52), significantly outperforming immersion ultrasound, which achieved the same target in only 72.2% (13/18) (p = 0.043). This difference is clinically meaningful and highlights the superiority of laser interferometry in long eyes, where ultrasound measurements are more prone to operator-dependent variability and axial compression errors. However, the relatively small number of high-myopia cases assessed with immersion biometry may have limited statistical robustness for subgroup comparisons. Laser interferometry is more accurate in the localization of the point of retinal fixation, even in the presence of posterior staphyloma [13,14]. In contrast, within the control group, laser interferometry and immersion biometry showed comparable refractive accuracy (p = 1.00), which is consistent with a previous study [15]. This suggests that in normal axial-length eyes, immersion biometry remains reliable, while in long eyes, laser interferometry should be the preferred method to minimize refractive surprises.

The high myopia group was intentionally targeted with a more negative preoperative targeted SE compared to the control group (p < 0.001). Many surgeons attempt to compensate for postoperative hyperopia by empirically targeting a moderately myopic postoperative refraction (-1.00D to -2.00 D) as described by Behunin and Pantanelli [16] and (-0.50 D to -2.00 D) as reported by Chong and Mehta [17]. This strategy helps compensate for potential biometry inaccuracies and variations in effective lens position in highly myopic eyes.

Despite these observed clinical patterns, logistic regression did not identify any statistically significant predictors of achieving postoperative SE within ±1.0D. High AXL showed a non-significant trend toward reduced refractive accuracy, which aligns with established evidence that long eyes (≥26 mm) are more prone to prediction error due to challenges in estimating effective lens position, posterior staphyloma, and formula limitations [18]. The lack of statistical significance in our study is likely related to the small number of refractive outliers. Nonetheless, the direction of effect supports the clinical relevance of AXL as a contributing factor. Larger studies with greater representation of extreme AXL may help clarify the predictors of refractive variability in long eyes.

Clinical implication

This study emphasizes the importance of using laser interferometry as the primary biometry method in highly myopic eyes to minimize refractive surprises. Clinicians should consider targeting a slightly more myopic refraction in long axial-length eyes to improve postoperative accuracy.

Limitations

This study was conducted in a real-world clinical setting where postoperative outcomes were obtained from multiple surgeons rather than a single operator, introducing potential variability in surgical technique and outcome measurement. In addition, as Penang is a Malaysian state with a higher proportion of Chinese ethnicity, the study population may not be representative of the broader national demographic. This ethnic distribution could contribute to the observed higher prevalence of high myopia among participants and may limit the generalizability of the findings to other populations. Finally, the number of high-myopia cases assessed with immersion biometry was relatively small, which may reduce the statistical power for subgroup comparison. The absence of statistically significant predictors in the logistic regression models is influenced by the low number of refractive outliers, which limits the ability to detect smaller effect sizes.

## Conclusions

High myopia was significantly associated with male sex and Chinese ethnicity, highlighting the importance of early detection in higher-risk populations. There was no significant difference in postoperative BCVA between the high myopia and control groups. Laser interferometry demonstrated significantly better refractive outcomes than immersion biometry in the high myopia group. In cases of high myopia, the preoperative targeted SE was targeted to be slightly more myopic than in the control groups in order to achieve comparable postoperative SE outcomes between the two groups, supporting its relevance in surgical planning for long AXL eyes.
